# A prospective randomized split-mouth trial comparing immediate versus early anterior implant placement with customized healing abutments: 12-month marginal bone loss, stability, and esthetic outcome

**DOI:** 10.3389/fdmed.2026.1884486

**Published:** 2026-07-29

**Authors:** Sara N. El Touliani, Ahmed M. Abdelhamid, Nada M. H. Fahmy, Yasser M. Aly

**Affiliations:** 1 Oral Rehabilitation Sciences Department, Faculty of Dentistry, Beirut Arab University, Beirut, Lebanon; 2 Faculty of Dentistry, Alexandria University, Alexandria, Egypt; 3 Faculty of Dentistry, Arab Academy for Science, Technology and Maritime Transport, Alexandria, Egypt

**Keywords:** anterior maxilla, customized healing abutment, early implant placement, immediate implant placement, marginal bone loss, pink esthetic score (PES)

## Abstract

**Objectives:**

To compare 12-month marginal bone loss (MBL) between immediate implant placement (IIP) and early implant placement (EIP) in the anterior region. Secondary objectives included the evaluation of implant stability (ISQ), esthetic outcomes (PES–WES), and peri-implant health (probing depth and bleeding index) when using customized healing abutments.

**Methods:**

A prospective, randomized, split-mouth clinical trial was conducted involving 12 patients, with a total of 24 implant sites. The protocol was approved by the institutional review board (IRB2023-H0113D-P-0494), registered at ClinicalTrials.gov (NCT0698551). Each patient received one IIP and one contralateral EIP via fully guided surgery. Chairside-fabricated customized healing abutments were utilized, and definitive restorations were delivered at 3 months. Outcomes were recorded at 3, 6, and 12 months. MBL was assessed via standardized CBCT, implant stability via resonance-frequency analysis, and esthetics through Pink/White Esthetic Scores. Mixed-effects models were used for statistical analysis (*α* = 0.05).

**Results:**

IIP demonstrated significantly lower early MBL at 3 months (mean difference: −0.30 mm, 95% CI: −0.52 to −0.08; *p* = 0.012) and 6 months (mean difference: −0.22 mm, 95% CI: −0.39 to −0.05; *p* = 0.016), and higher ISQ at 3 months (mean difference: 2.49, 95% CI: 0.49–4.49; *p* = 0.017) compared to EIP. However, no significant differences were observed in MBL (mean difference: −0.01 mm, 95% CI: −0.08 to 0.06; *p* = 0.618) or ISQ (mean difference: 0.20, 95% CI: −1.62 to 2.02; *p* = 0.823) at the 12-month primary endpoint. Regarding esthetics, total PES favored IIP at 6 months (mean difference: 2.09, 95% CI: 1.48–2.70; *p* < 0.001) and 12 months (mean difference: 1.50, 95% CI: 0.92–2.08; *p* < 0.001). WES was similar at 6 months (mean difference: 0.42, 95% CI: −0.31 to 1.15; *p* = 0.234) but favored IIP at 12 months (mean difference: 1.00, 95% CI: 0.50–1.50; *p* = 0.002). Peri-implant health parameters improved over time in both groups with no implant losses.

**Conclusions:**

When combined with guided protocols and customized healing abutments, IIP and EIP yield comparable 12-month marginal bone outcomes. IIP provides an advantage in accelerated early stability and superior soft-tissue esthetic scores (PES) compared to EIP. The clinically meaningful esthetic advantages of IIP support its preferential use in the anterior zone when anatomical conditions permit.

## Introduction

The replacement of compromised anterior teeth with dental implants is a widely accepted and predictable approach for restoring function and esthetics. In contemporary implant dentistry, the focus has shifted from mere survival rates to the long-term preservation of peri-implant hard and soft tissues and the harmonious esthetic integration of the final restoration. Two critical factors influencing these outcomes are the timing of implant placement and the configuration of the transmucosal components, specifically the use of customized healing abutments. These devices, which can be fabricated from materials such as titanium or zirconia, are essential for facilitating soft-tissue adaptation and maintaining a natural emergence profile (1–3).

Predictable success in the esthetic zone requires a high level of precision, often achieved through fully guided surgery and digital scanning technologies. Such workflows allow clinicians to meticulously plan the implant trajectory and depth, ensuring the appropriate distance is maintained between the restorative margin and the marginal bone to prevent biological complications. Furthermore, the choice of the final prosthesis—whether screw-retained or cement-retained—plays a significant role in the clinical maintenance and esthetic outcome of the case (4–6).

The soft-tissue profile and peri-implant healing are critical determinants of success in the smile zone. Custom-made abutments tailored to individual patient anatomy can significantly improve surgical outcomes by providing immediate peri-implant tissue support and shaping a natural emergence profile. This conditioning prepares the site for the definitive restoration and helps reduce the volumetric contour changes often seen with stock components (7–9).

Despite these advancements, there is ongoing discussion regarding the optimal timing for implant placement—immediate (IIP) vs. early (EIP)—when combined with customized healing protocols. While IIP offers the advantage of reduced treatment time, EIP may allow for additional soft-tissue healing. There is a need for high-quality evidence to compare these protocols regarding their impact on marginal bone loss (MBL) and esthetic scores (PES/WES) (10, 11).

Accordingly, the aim of the present randomized split-mouth clinical trial was to evaluate the effect of customized healing abutments on MBL and peri-implant soft-tissue esthetics in IIP and EIP protocols. The null hypothesis was that there would be no significant difference between IIP and EIP at 12 months for marginal bone loss; secondary outcomes included implant stability (ISQ), esthetic scores (PES/WES), and peri-implant health parameters (PD/BI).

## Material and methods

### Study design and setting

This prospective, randomized, split-mouth clinical trial compared immediate implant placement (IIP) with early implant placement (EIP) in the anterior maxilla under a unified, prosthetically driven digital workflow with customized healing abutments. Each participant contributed two homologous sites: one allocated to IIP and the contralateral site to EIP. The study was conducted at the Department of Prosthodontics, Faculty of Dentistry, Beirut Arab University, Beirut, Lebanon, between January 2023 and December 2024. Patient recruitment and all surgical procedures were performed at the university-based prosthodontic clinic.

**Figure 1:**
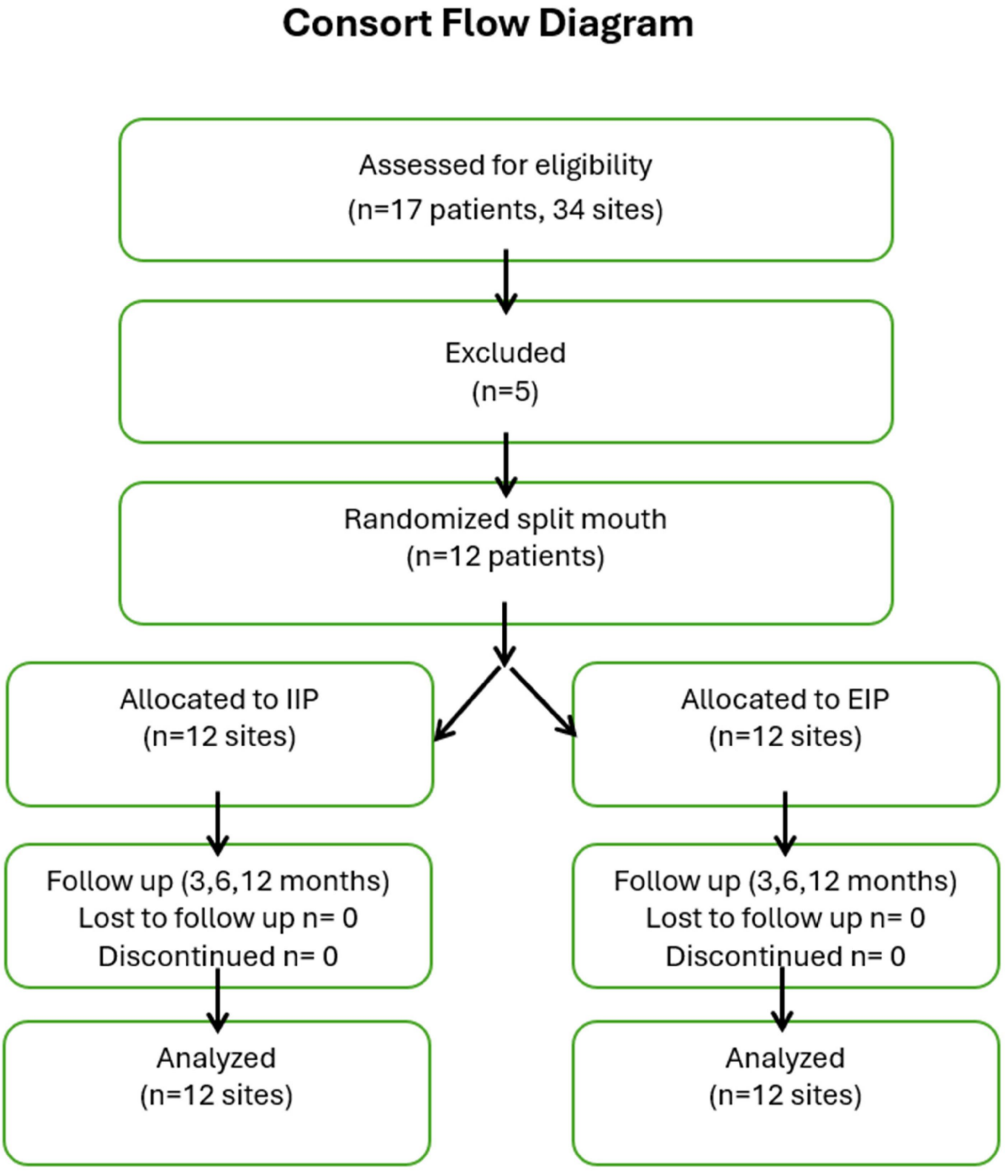
CONSORT flow diagram of participant screening, allocation, follow**-**up, and analysis.

The protocol was approved by the institutional review board (IRB2023-H0113D-P-0494), registered at ClinicalTrials.gov (NCT0698551), and conducted in accordance with the Declaration of Helsinki; a written informed consent was obtained from all participants. The CONSORT diagram is presented in Figure 1. Timing strategies were defined following contemporary guidance for esthetic sites (immediate at extraction vs. early after short soft tissue healing), balancing risks to the labial plate, phenotype, and esthetic expectations. A fully guided approach was used to standardize three-dimensional implant positioning and minimize surgical variability in the esthetic zone (11, 12).

### Participants

Participants were recruited from a university-based prosthodontic clinic. During the study period (January 2023 to December 2024), 47 patients with bilateral unrestorable maxillary anterior teeth were assessed for eligibility at our clinical center. Of these, 17 were screened for detailed evaluation, and 12 met all inclusion criteria and were enrolled. The remaining 35 patients were excluded for the following reasons: 18 had insufficient bone volume (<4 mm apical bone or <2 mm labial plate), 8 had active periodontal or endodontic infection, 5 had thin soft-tissue phenotype, 2 were heavy smokers, 1 had uncontrolled systemic disease, and 1 declined participation.

#### Inclusion criteria

- Adults aged 20–40 years
- Requirement for replacement of bilateral, unrestorable maxillary anterior teeth
- Harmonious gingival contours
- Thick soft-tissue phenotype (≥2 mm)
- Radiographic prerequisites: ≥4 mm of apical bone beyond the root apex and ≥2 mm labial plate thickness for each site
- No periapical radiolucency
- Complete and accessible medical and dental records

#### Exclusion criteria

- Systemic contraindications to surgery or osseointegration
- Active periodontal or endodontic infection
- Dehiscence/fenestration at baseline
- Poor oral hygiene
- Heavy smoking (>10 cigarettes/day)
- Uncontrolled parafunction

These criteria aimed to reduce confounders known to influence hard/soft-tissue stability and esthetic outcomes in anterior implants.

### Sample size calculation

Sample size was calculated *a priori* using G*Power 3.1.9.2 (Heinrich Heine University, Düsseldorf, Germany) for a two-tailed paired *t*-test (reflecting the split-mouth design). Based on previous systematic reviews of immediate vs. delayed implant placement (13), a clinically meaningful difference of 0.25 mm in marginal bone loss (MBL) was considered the minimum detectable difference. Assuming a standard deviation of 0.20 mm (based on pilot data and published literature), an alpha of 0.05, and 80% power, the required sample size was 10 patients per group. Accounting for a 20% potential attrition rate, 12 patients (24 sites) were enrolled to ensure adequate power for the primary endpoint analysis. This sample size also provided sufficient power (≥80%) for detecting a moderate effect size (Cohen's *d* = 0.8) in the primary outcome using the paired design.

### Randomization and allocation

Within each patient, random allocation to IIP/EIP sides was generated by computer and concealed in sealed opaque envelopes opened at surgery (10). The split-mouth design reduces intersubject variability for timing comparisons while preserving clinical generalizability.

### Preoperative assessment

**Figure 2:**
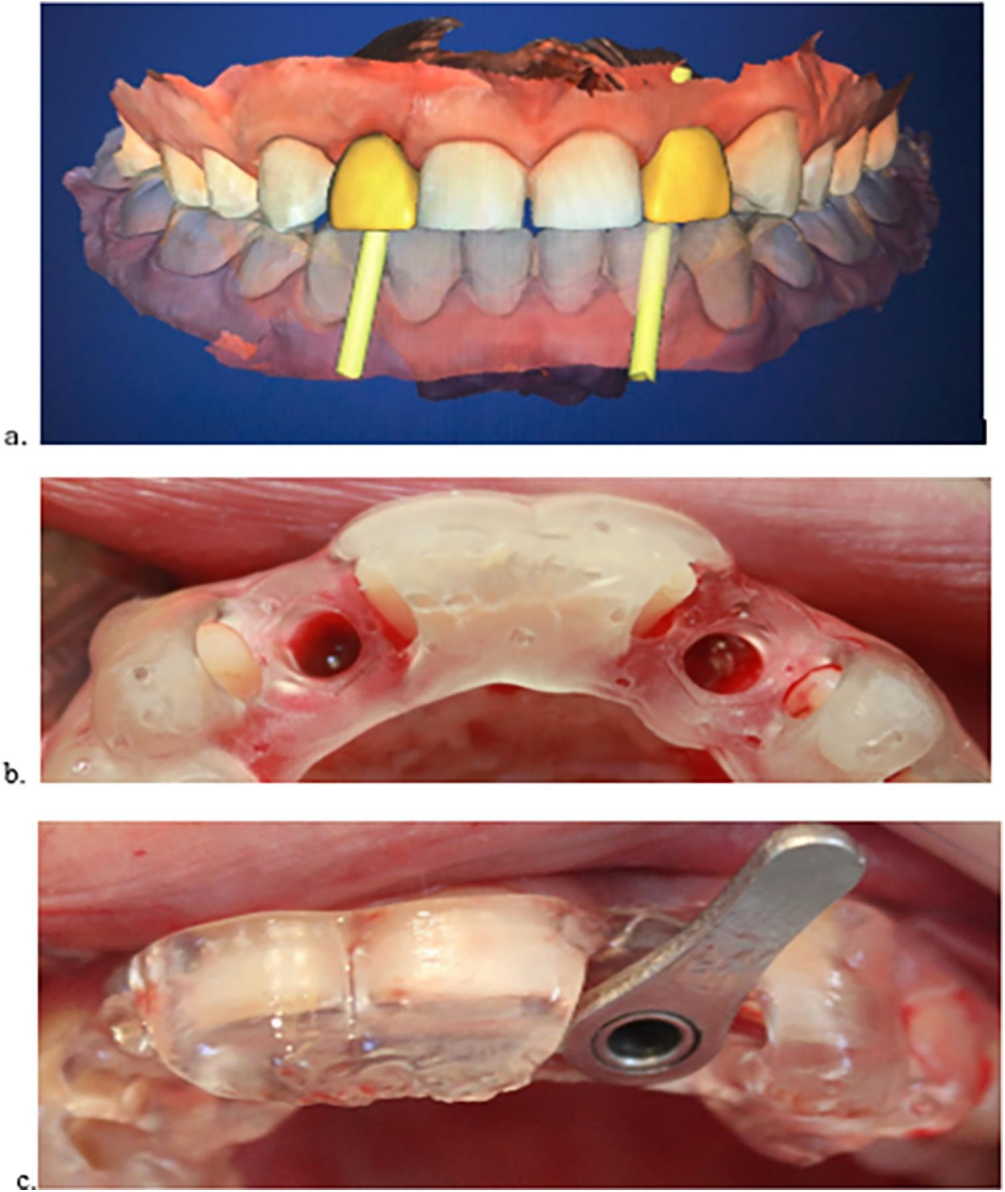
Representative clinical sequence: extraction and immediate implant placement. **(a)** Virtual designing for implant planning, **(b)** surgical guide in place, **(c)** guide spoon.

Preoperative assessment included standardized clinical photographs, periodontal charting, and cone-beam computed tomography (CBCT). Diagnostic models or intraoral scans were merged with CBCT for prosthetically driven planning; virtual implant positioning prioritized a palatal entry point, correct buccolingual trajectory, and apico-coronal depth compatible with the planned emergence profile (Figure 2a). Static guides were fabricated and verified clinically prior to osteotomy to ensure accurate seating. CBCT-based planning and guided surgery have been advocated to improve positional accuracy and risk control in the anterior maxilla (12, 14).

### Surgical protocol

All participants received a systemic antibiotic (2 g amoxicillin-clavulanic acid, per-orally). All procedures were performed under local anesthesia (4% articaine). All extractions were performed atraumatically using periotomes and luxators to preserve the integrity of the socket walls, particularly the buccal plate. Flap elevation was avoided whenever possible.

Implant osteotomies were prepared using a fully guided surgical protocol according to the manufacturer's instructions, ensuring prosthetically driven positioning. Primary stability was achieved through apical anchorage beyond the extraction socket, and implant placement torque was recorded.

#### Immediate implant placement (IIP)

Atraumatic extraction and implant (IDI Implant Diffusion International) atraumatic extraction and implant placement were performed in the same visit using the surgical guide (Figures 2b,c).

#### Early implant placement (EIP)

The corresponding tooth was extracted, and implant placement was performed 4 weeks later following soft-tissue closure. The rationale for this interval was to permit soft-tissue closure and defect appraisal while preserving ridge form for esthetic contouring.

#### Buccal gap management

For IIP sites, the buccal gap (jumping distance) between the implant surface and the socket wall was measured intraoperatively. In all IIP sites, the gap was ≤2 mm and was left to heal spontaneously without grafting, as primary stability was achieved through apical engagement and the gap was considered clinically acceptable. For EIP sites, the extraction sockets were allowed to heal with blood clot preservation for 4 weeks. No guided bone regeneration or contour augmentation was performed in any EIP site, as the inclusion criteria required ≥2 mm labial plate thickness and adequate ridge dimensions. This standardized approach (no grafting in either group) ensured that the comparison between IIP and EIP was not confounded by differential use of graft materials or membranes.

Across both strategies, osteotomy preparation and implant insertion followed the same guided sequence (IDI surgical guided kit) and implant system to harmonize position and angulation.

### Customized healing abutments

**Figure 3:**
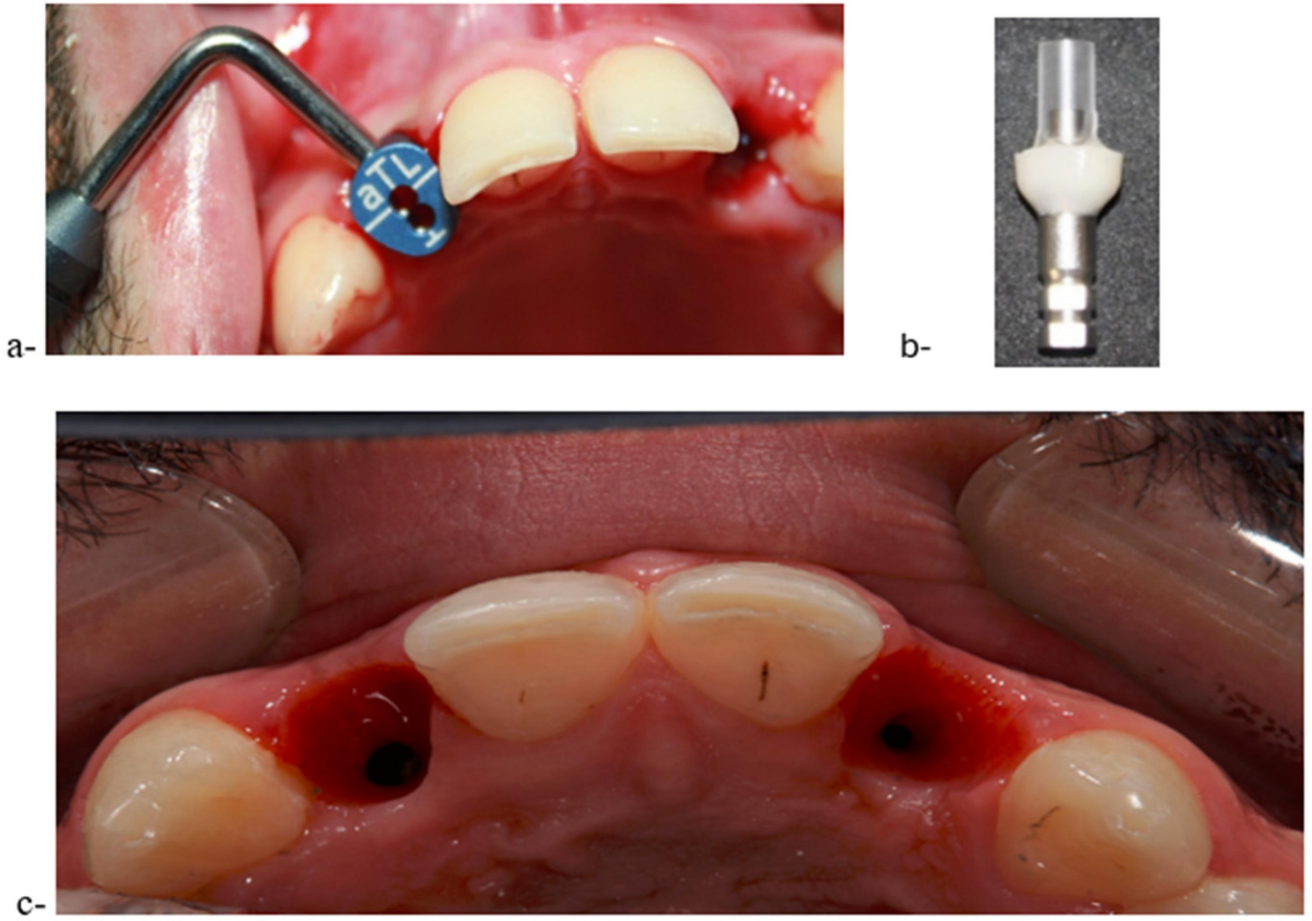
Clinical sequence of customized healing abutment. **(a)** Mesiodistal dimension using cervico guide, **(b)** customized abutment using cervico kit, **(c)** Emergence profile conditioning using a customized healing abutment and cervico template).

Immediately after implant placement, customized healing abutments were fabricated chairside using the Cervico Kit (Ariston Dental) and a nano-hybrid flowable composite (Filtek Supreme Flowable Composite, 3M) to reproduce the planned transmucosal contour; abutments were adjusted out of occlusion and torqued per manufacturer recommendations (Figures 3a,b).

### Restorative protocol

**Figure 4:**
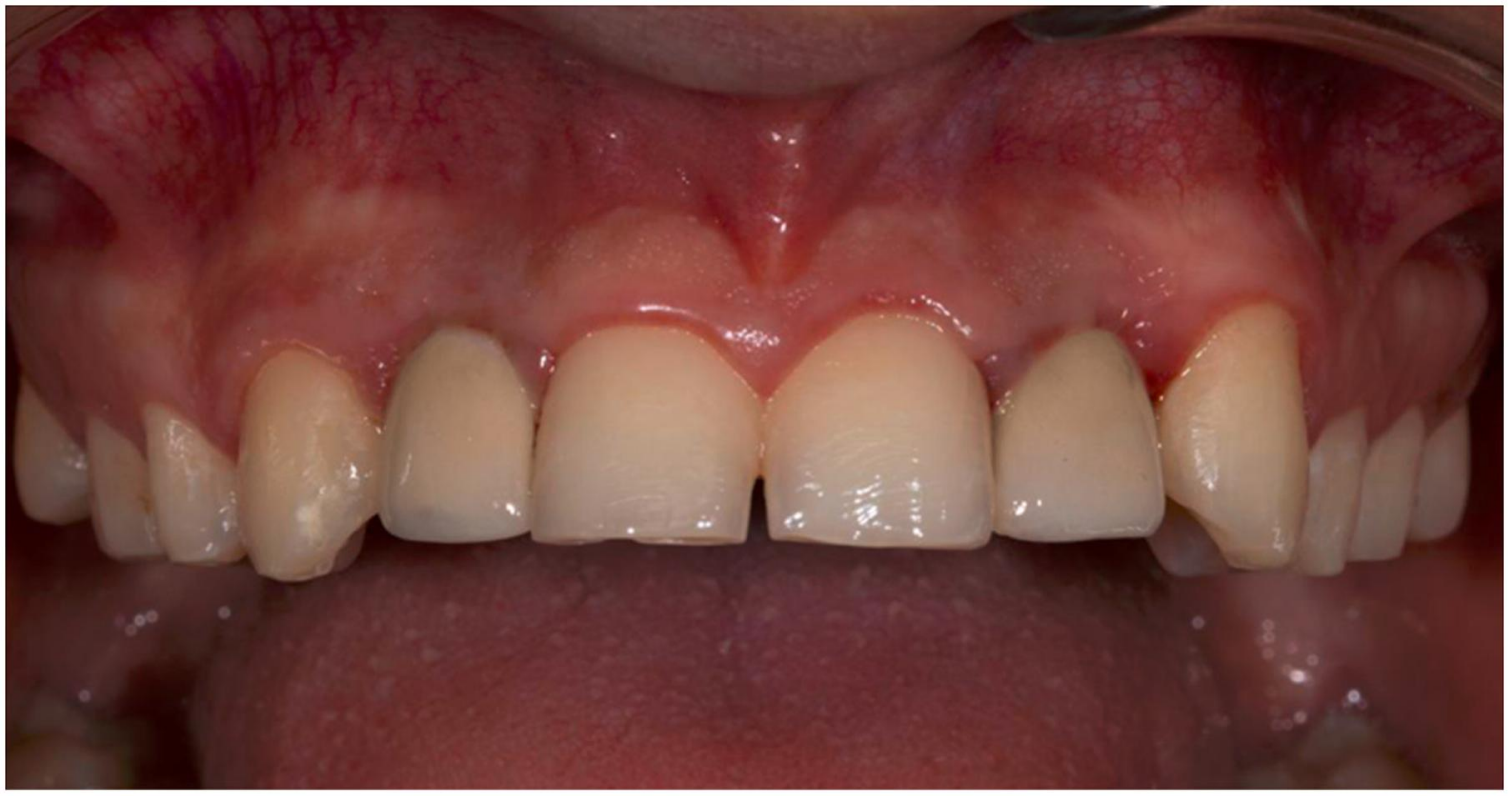
Definitive screw**-**retained zirconia restorations at 12 months (IIP and EIP): frontal smile and intraoral views.

**Figure 5:**
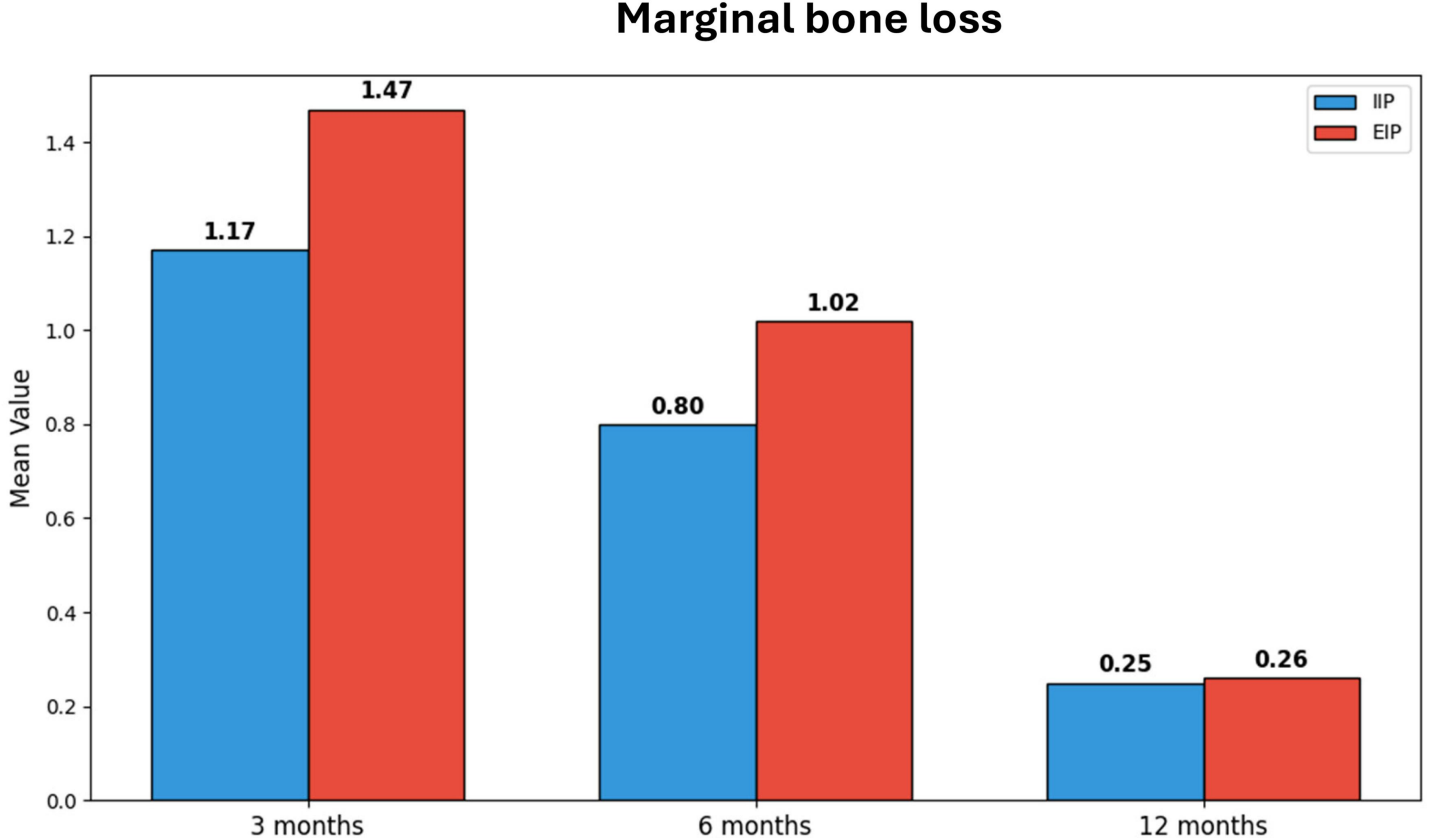
Marginal bone loss (MBL, mm) over time at 3, 6, and 12 months for immediate (IIP) vs. early (EIP) implant placement.

**Figure 6:**
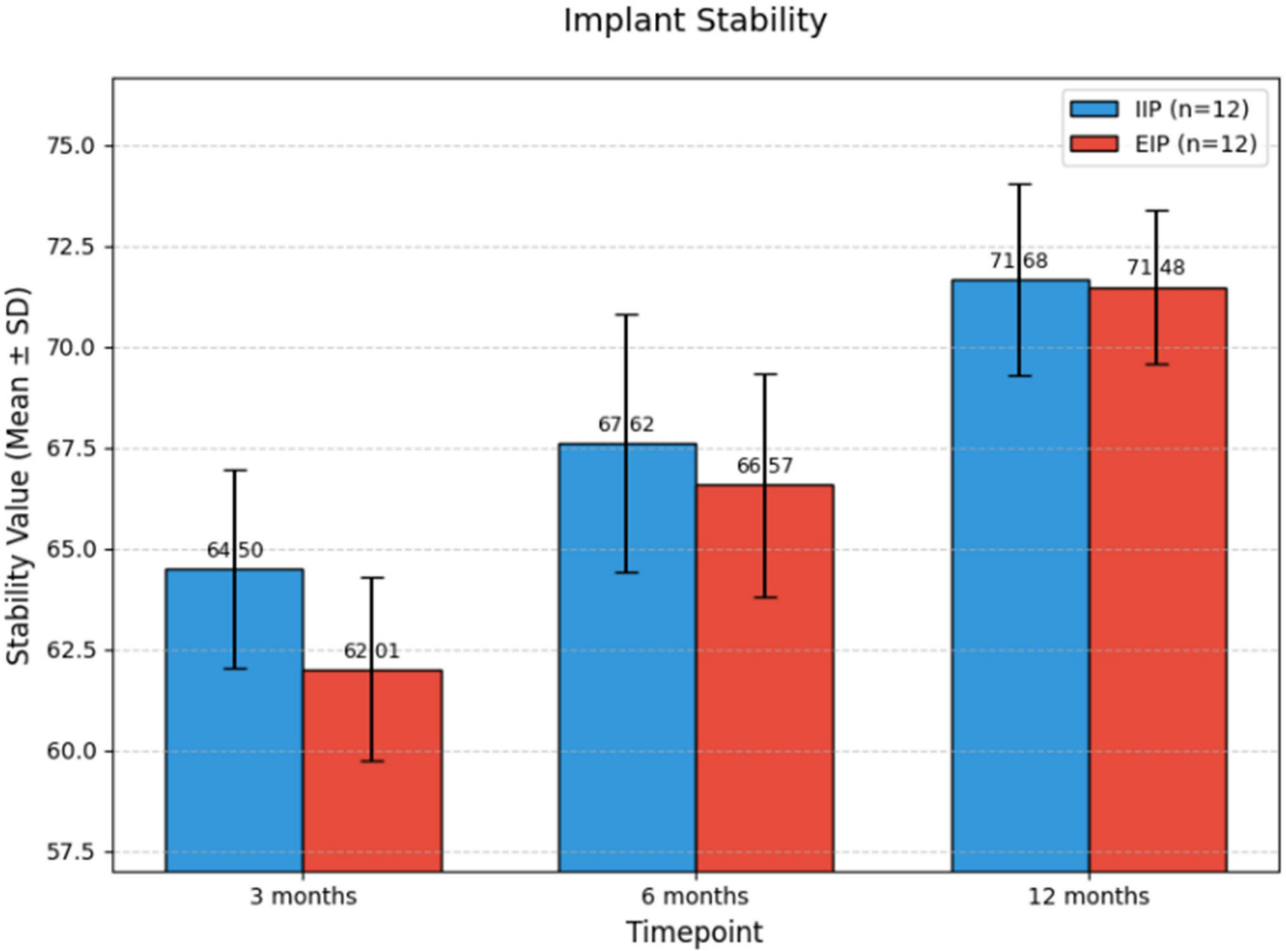
Implant stability quotient (ISQ) over time at 3, 6, and 12 months for immediate (IIP) vs. early (EIP) implant placement.

**Figure 7:**
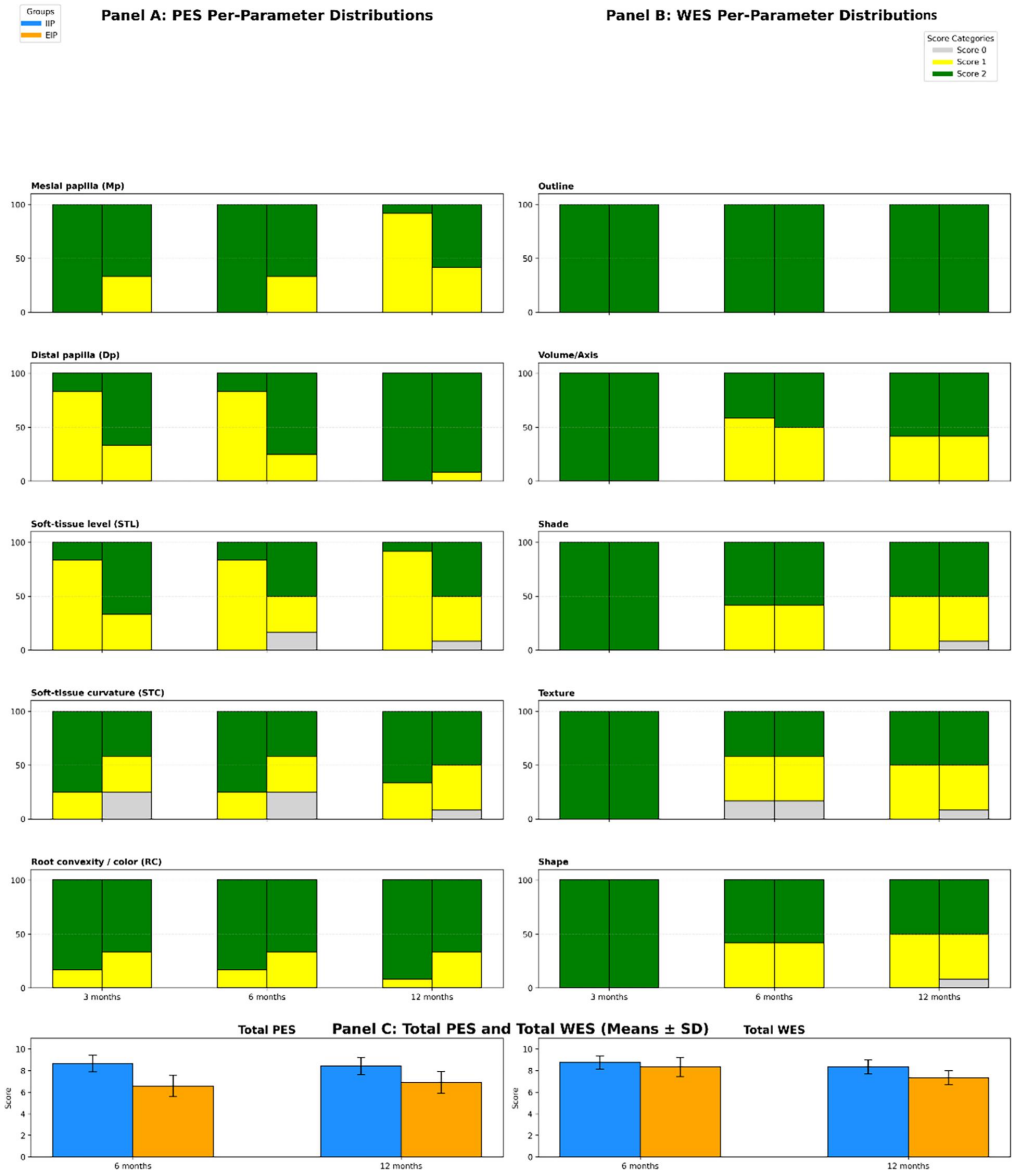
Pink (PES) and white (WES) esthetic scores at 3, 6, and 12 months for immediate (IIP) vs. early (EIP) implant placement. **(A)** PES per-parameter score distributions (score 0, 2, 2) for mesial papilla, distal papilla, soft-tissue level, soft-tissue curvature, and root convexity/color. **(B)** WES per parameter score distributions (score 0, 1, 2) for outline, volume/axis, shade, texture, and shape. **(C)** Total PES and WES (mean ± SD) at 6 and 12 months for IIP and EIP. *p < 0.05 after adjustment.

**Figure 8:**
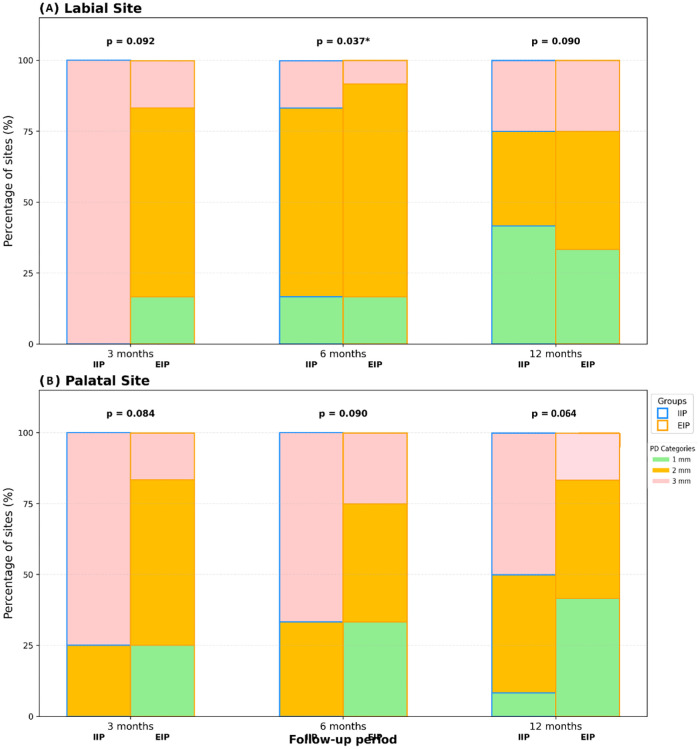
Probing depth (PD) frequency distributions (1, 2, and 3 mm) at labial and palatal sites at 3, 6, and 12 months for immediate (IIP) vs. early (EIP) implant placement. **(A)** Labial site distributions. **(B)** Palatal site distributions. Each stacked bar represents the percentage of sites (*n* = 12 per group) with PD of 1, 2, or 3 mm. *p < 0.05.

**Figure 9:**
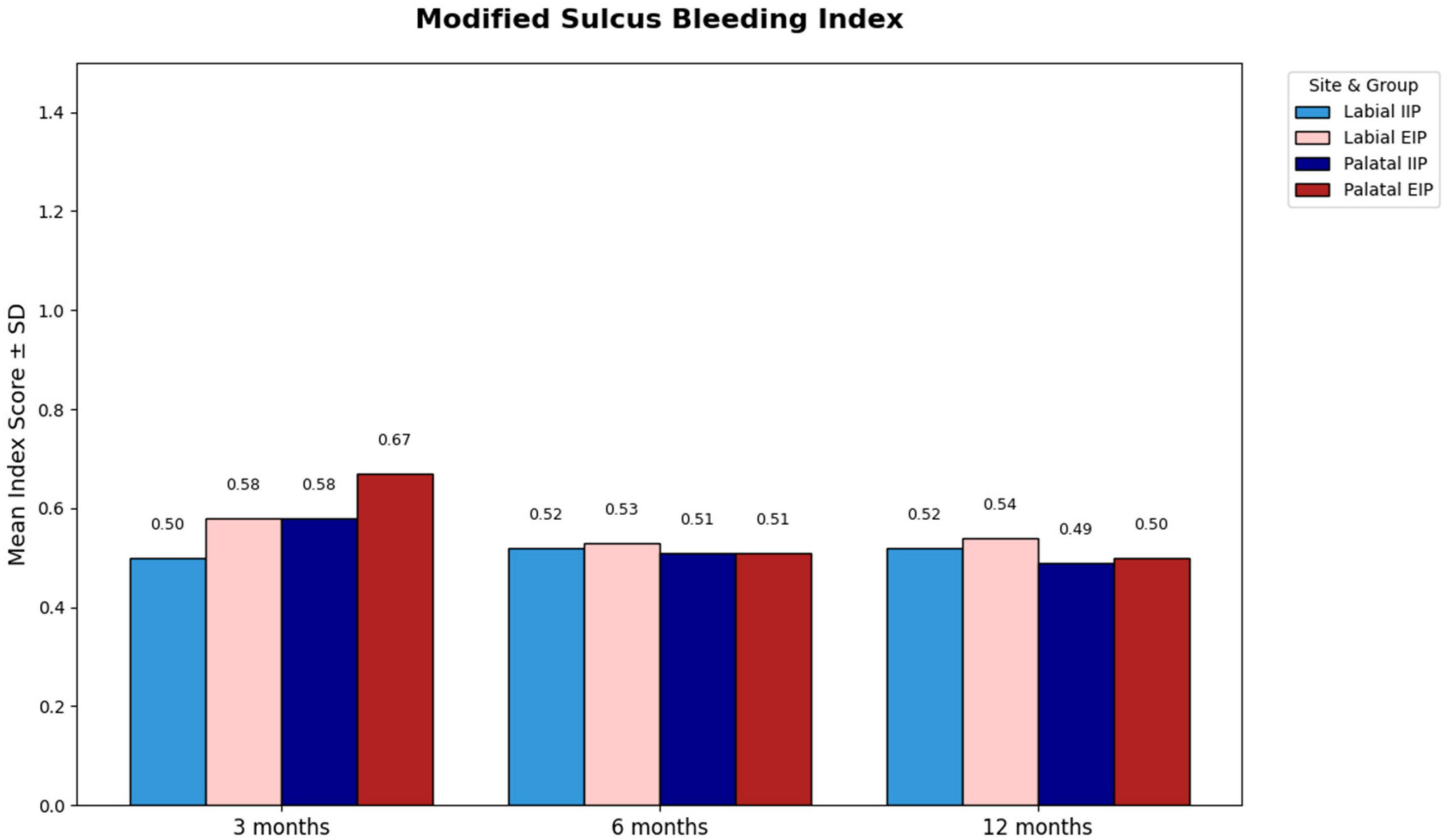
Modified sulcus bleeding index (BI) means at 3, 6, and 12 months (IIP vs. EIP).

The restorative workflow was standardized for both groups: at 3 months post-placement, healing abutments were removed and implant-level digital impressions were made with scan bodies (Figure 3c); crowns were CAD-designed (Exocad) and fabricated in monolithic/micro-layered zirconia, cemented extraorally to titanium bases, and screw-retained to a standardized torque (25 N cm) with screw-access hole and occlusion was adjusted to mutually protected articulation as recommended Figure 4. Representative clinical steps and measurement panels appear in Figures 5–7 (MBL, ISQ, and esthetic scoring) and Figures 8, 9 (probing depth and bleeding indices), with additional clinical documentation (14, 15).

### Outcome measures

Outcomes and measurement timing are summarized in Table 1. The primary endpoint was the between-group difference in MBL at 12 month follow up. Secondary endpoints included implant stability (ISQ), esthetic outcomes (PES/WES), and peri-implant health parameters such as probing depth (PD) and bleeding index (BI). These outcomes were assessed at 3, 6, and 12 months.

**Table 1:** Outcome measures, instruments, timepoints, assessor, and calibration.

Outcome	Definition/Units	Instrument/Method	Timepoints	Assessor (role)	Calibration/Standardization notes
Marginal bone loss (MBL)	Distance from implant reference (platform/neck) to first bone–implant contact; mm (mesial, distal; averaged)	Standardized CBCT; fixed FOV & voxel size; identical patient positioning; calibrated measurement software	3, 6, 12 months	Radiologist/Calibrated examiner	Same scanner/settings each visit; replicate measures for 10% of scans; mesial/distal averaged per implant
Implant stability (ISQ)	Resonance-frequency analysis; ISQ units (0–100)	RFA device with system-matched SmartPeg; three readings averaged	3, 6, 12 months	Surgeon/Calibrated examiner	Peg orientation perpendicular; device warm-up; triplicate readings averaged
Esthetics (PES)	Pink esthetic score (5 parameters, 0–2 each; total 0–10)	Standardized frontal photos (DSLR, macro, twin flash); contralateral reference/template	3, 6, 12 months	Prosthodontist + Periodontist (independent)	Two scoring sessions 1 week apart; calibration set; consensus if disagreement
Esthetics (WES)	White esthetic score (5 parameters, 0–2 each; total 0–10)	Same photo set as PES; shade tabs for color control	3, 6, 12 months	Prosthodontist + Periodontist (independent)	As above
Probing depth (PD)	mm; labial & palatal sites; integer mm (score levels 1–3 mm)	Plastic periodontal probe; light force	Baseline (crown insertion), 3, 6, 12 months	Periodontist/Calibrated examiner	Force-standardized; same probe model; duplicate checks in 10% of sites
Bleeding index (BI)	0–3 (0 none; 1 points; 2 line; 3 profuse)	Bleeding on gentle probing at labial/palatal; averaged per implant	Baseline (crown insertion), 3, 6, 12 months	Periodontist/Calibrated examiner	Scored within 30 s of probing; site-level values averaged per implant

#### Marginal bone loss (MBL)

MBL was quantified on standardized CBCT datasets at 3, 6, and 12 months using identical field of view (5 cm × 5 cm), voxel size (0.2 mm), and patient positioning. CBCT scans were performed using a standardized protocol with fixed field of view, voxel size, and identical patient positioning for all timepoints. Tube settings were 90 kVp and 8 mA. All scans were acquired using the same CBCT device (Planmeca ProMax 3D, Planmeca, Helsinki, Finland). Measurements from a reproducible implant reference (platform/neck) to the first bone-to-implant contact were recorded mesially and distally and averaged per implant to 0.01 mm. Standardized CBCT-based dimensional evaluation with consistent geometry helps minimize systematic error in longitudinal bone measurements.

#### Measurement reliability

MBL measurements were performed by a single calibrated oral radiologist. Intra-examiner reliability was assessed by repeating measurements on 20% of scans 1 month apart; the intraclass correlation coefficient (ICC) was 0.94 (95% CI: 0.90–0.97). Inter-examiner reliability between two calibrated radiologists was also excellent (ICC = 0.92, 95% CI: 0.87–0.95). The standard error of measurement was 0.04 mm, and the minimal detectable change was 0.11 mm. MBL was measured from the implant platform/neck reference point at the time of final restoration delivery (3 months) as baseline. Subsequent measurements at 6 and 12 months represented changes from this baseline reference. A decrease in the measured value over time indicates bone maturation and remodeling rather than “reversal of bone loss.” The term “marginal bone level change” is used to reflect this dynamic process.

#### Radiation justification

CBCT was selected for MBL assessment to enable three-dimensional evaluation of bone changes around implants, which is not possible with two-dimensional periapical radiography. The use of CBCT allowed consistent patient positioning, standardized measurement planes, and accurate assessment of buccal and lingual bone changes. The radiation dose per scan was minimized using a reduced FOV (5 cm × 5 cm) and low-dose protocol (effective dose: ∼8 μSv), which is comparable to a series of periapical radiographs. All scans were performed using the same device and parameters to ensure consistency (16, 17).

#### Implant stability (ISQ)

Resonance-frequency analysis (ISQ) provides a non-invasive measure of implant stability over time, allowing longitudinal assessment of osseointegration was recorded by resonance-frequency analysis (Ostell) with system-matched SmartPegs; three perpendicular readings were averaged per site/timepoint. RFA provides a validated, non-invasive index of time-dependent implant stability and is widely used in timing trials.

#### Esthetic evaluation

Esthetics were evaluated on standardized frontal intraoral photographs at 3, 6, and 12 months (DSLR, macro lens, twin flash, cheek retractors, shade tabs for white balance). PES comprised five soft-tissue parameters (mesial/distal papilla, soft-tissue level/curvature, root convexity/soft-tissue color), each scored 0–2; WES rated five crown-related parameters (outline, volume/axis, color/shade, surface texture, translucency) on the same 0–2 scale. Two calibrated evaluators (prosthodontist, periodontist) scored images independently in two sessions 1 week apart; discrepancies were resolved by consensus.

#### Reliability of esthetic assessments

Inter-rater reliability for PES and WES assessments was evaluated using intraclass correlation coefficients (ICC, two-way random effects model, absolute agreement) with 95% confidence intervals. Inter-rater reliability was excellent for total PES (ICC = 0.91, 95% CI: 0.86–0.94) and WES (ICC = 0.89, 95% CI: 0.84–0.93). Intra-rater reliability was good to excellent for PES (Evaluator 1: ICC = 0.92, 95% CI: 0.88–0.95; Evaluator 2: ICC = 0.90, 95% CI: 0.85–0.94) and WES (Evaluator 1: ICC = 0.88, 95% CI: 0.82–0.92; Evaluator 2: ICC = 0.87, 95% CI: 0.81–0.91).

#### Peri-implant health parameters

Probing depth was recorded at labial and palatal aspects with a plastic probe and light force (integer millimeters; reported as 1–3 mm categories for frequency tables), and BI was scored on a 0–3 ordinal scale (0 = no bleeding; 1 = isolated points; 2 = confluent line; 3 = profuse). Maintenance included personalized oral-hygiene instruction and professional prophylaxis at 1, 3, 6, and 12 months.

### Bias control

To minimize bias, one experienced operator performed all surgeries using the same guided protocol and implant system; one prosthodontist delivered all prosthetic procedures. Radiographic and esthetic assessors followed fixed operating procedures and underwent calibration with pilot cases. Data were recorded on electronic case-report forms and verified against source documents; protocol deviations (missed visits, imaging artifacts, unplanned adjustments) were documented before data lock.

### Statistical analysis

Statistical analyses were conducted using IBM SPSS Statistics (version 26.0, IBM Corp., Armonk, NY, USA) and R version 4.2.1 (R Foundation for Statistical Computing, Vienna, Austria). Mixed-effects models were implemented via the lme4 package in R, and sample size calculations utilized G*Power (version 3.1.9.2). The Shapiro–Wilk test assessed the normality of continuous variables, with parametric tests applied to normally distributed data. Non-normal data underwent transformations or used non-parametric tests, such as the Wilcoxon signed-rank test for paired comparisons and the Friedman test for repeated measures. Continuous data were summarized as mean ± standard deviation (SD) or median (interquartile range, IQR) based on their distribution.

The primary endpoint, 12-month marginal bone loss (MBL), was analyzed using a paired *t*-test at *α* = 0.05. Linear mixed-effects models were fitted for secondary outcomes, incorporating fixed effects for group, time, and their interaction, with a random intercept for each patient. Model assumptions were validated, and multiplicity was controlled using the Holm-Bonferroni method, with Tukey's HSD test for *post-hoc* comparisons.

Categorical data were analyzed using McNemar's test for 2 × 2 tables and the Stuart-Maxwell test for more than two categories, calculating odds ratios and 95% confidence intervals. Effect sizes were determined using Cohen's *d* (with Hedges' *g* correction) and categorized as small, moderate, or large. A two one-sided test (TOST) procedure assessed clinical equivalence for the primary endpoint with an equivalence margin of ±0.25 mm. All tests were two-tailed with significance set at *α* = 0.05, and *p*-values were reported to three decimal places, with values <0.001 denoted as *p* < 0.001.

## Results

### Participant flow and baseline characteristics

Seventeen individuals were screened for eligibility, of whom twelve met the inclusion criteria and were enrolled in the study (24 implant sites). Participant flow is summarized in Figure 1. All planned evaluations at 3, 6, and 12 months were obtained with no implant losses or major complications recorded during the observation period. All 12 enrolled patients completed the 12-month follow-up period (100% retention rate). Complete outcome data were available for all 24 implant sites at all timepoints (3, 6, and 12 months) for all outcome measures, including MBL, ISQ, PES, WES, PD, and BI. Therefore, no imputation for missing data was required.

### Baseline characteristics

Baseline site characteristics were well-balanced between IIP and EIP groups (Table 1). There were no significant differences between groups in tooth type distribution (central vs. lateral incisors; *p* = 0.721, Fisher's exact test), pre-extraction labial plate thickness (IIP: 2.34 ± 0.42 mm; EIP: 2.28 ± 0.38 mm; *p* = 0.681, paired *t*-test), or bone density as measured by CBCT Hounsfield units (IIP: 612.3 ± 78.5 HU; EIP: 605.8 ± 82.1 HU; *p* = 0.742, paired *t*-test). Insertion torque values were similar between groups (IIP: 38.2 ± 4.6 Ncm; EIP: 36.9 ± 5.1 Ncm; *p* = 0.388, paired *t*-test). All patients had a thick soft-tissue phenotype (≥2 mm) as determined by clinical assessment. Graphical summaries of the longitudinal outcomes are presented in Figures 5–9 with representative clinical documentation in Figures 2–4.

### Marginal bone loss (MBL)

The primary endpoint, 12-month marginal bone level change, was comparable between strategies (0.25 ± 0.08 mm for IIP and 0.26 ± 0.05 mm for EIP; mean difference: −0.01 mm, 95% CI: −0.08 to 0.06; *p* = 0.618, paired *t*-test) (Figure 5). The effect size (Cohen's *d*) was 0.15 (95% CI: −0.65 to 0.95), indicating a small effect. The 95% confidence interval for the mean difference did not include the clinically meaningful threshold of 0.25 mm, suggesting that the observed difference is not clinically significant.

#### Equivalence testing

The two one-sided test (TOST) procedure confirmed equivalence between IIP and EIP for 12-month MBL, as the 90% confidence interval for the mean difference (−0.07 to 0.05 mm) fell entirely within the pre-specified equivalence bounds of ±0.25 mm (*p*_equivalence = 0.008). This provides statistical evidence that the two-timing strategies produce clinically comparable MBL outcomes at 1 year.

#### Longitudinal analysis

Linear mixed-effects modeling revealed a significant main effect of time [*F*_(2, 44)_ = 342.6, *p* < 0.001], a significant group × time interaction [*F*_(2, 44)_ = 4.8, *p* = 0.014], but no significant main effect of group [*F*_(1, 11)_ = 1.2, *p* = 0.296]. The significant interaction indicates that the rate of marginal bone level change over time differed between the two strategies, with IIP showing lower early bone loss followed by convergence by 12 months. *post-hoc* pairwise comparisons (Tukey's HSD, *α* = 0.05) confirmed significant reductions in marginal bone levels from 3 to 6 months and from 6 to 12 months within both groups (all *p* < 0.001), consistent with early remodeling followed by stabilization (Table 2A).

**Table 2A:** Comparison of marginal bone loss (MBL, mm) between immediate and early implant placement from final restorations.

Follow-up	IIP (*n* = 12) mean ± SD	EIP (*n* = 12) mean ± SD	Mean difference (95% CI)	*p* value**	Effect size (Cohen's *d*, 95% CI)
3 months	1.17 ± 0.26ᵃ	1.47 ± 0.28ᵃ	−0.30 (−0.52 to −0.08)	0.012*	1.12 (0.26 to 1.98)
6 months	0.80 ± 0.16ᵇ	1.02 ± 0.25ᵇ	−0.22 (−0.39 to −0.05)	0.016*	1.04 (0.18 to 1.90)
12 months	0.25 ± 0.08ᶜ	0.26 ± 0.05ᶜ	−0.01 (−0.08 to 0.06)	0.618	0.15 (−0.65 to 0.95)
*p* value (within group, over time)	<0.001*	<0.001*			

Different superscript lowercase letters (ᵃ, ᵇ, ᶜ) denote statistically significant differences between follow-up points within the same group (Bonferroni-adjusted *p* < 0.0167).

* Statistically significant at adjusted *α* = 0.05.

** Adjusted using Holm-Bonferroni correction for the three between-group comparisons at 3, 6, and 12 months.

Between groups, IIP showed a more favorable early profile: lower marginal bone level changes at 3 months (1.17 ± 0.26 vs. 1.47 ± 0.28 mm; mean difference: −0.30, 95% CI: −0.52 to −0.08; *p* = 0.012) and at 6 months (0.80 ± 0.16 vs. 1.02 ± 0.25 mm; mean difference: −0.22, 95% CI: −0.39 to −0.05; *p* = 0.016). Prespecified pairwise contrasts confirmed significant improvements within each arm at all tested intervals (Table 2B), supporting the time-dependent convergence to similar 12-month levels.

**Table 2B:** Pairwise comparison regarding CBL (mm) within each group.

Comparison	IIP *p*-value	EIP *p*-value
3 vs. 6 months	0.001*	0.001*
3 vs. 12 months	<0.001*	<0.001*
6 vs. 12 months	<0.001*	<0.001*

* Adjusted using Bonferroni correction (*α* = 0.05/3 = 0.0167). Statistically significant at *p* < 0.0167.

### Implant stability (ISQ)

Implant stability increased steadily across the follow-up in both protocols (within-group *p* < 0.0001), with IIP demonstrating higher stability at the 3-month assessment (IIP 64.50 ± 2.46 vs. EIP 62.01 ± 2.27 ISQ, mean difference: 2.49, 95% CI: 0.49–4.49; *p* = 0.017), and numerical convergence by 6 months (67.62 ± 3.19 vs. 66.57 ± 2.75, mean difference: 1.05, 95% CI: −1.45 to 3.55; *p* = 0.393) and 12 months (71.68 ± 2.38 vs. 71.48 ± 1.91, mean difference: 0.20, 95% CI: −1.62 to 2.02; *p* = 0.823) as shown in (Table 3A, Figure 6).

**Table 3A:** Comparison of implant stability (ISQ) between immediate and early implant placement from final restorations.

Follow-up	IIP (*n* = 12) mean ± SD	EIP (*n* = 12) mean ± SD	Mean difference (95% CI)	*p* value**	Effect size (Cohen's *d*, 95% CI)
3 months	64.50 ± 2.46ᵃ	62.01 ± 2.27ᵃ	2.49 (0.49 to 4.49)	0.017*	1.05 (0.20 to 1.90)
6 months	67.62 ± 3.19ᵇ	66.57 ± 2.75ᵇ	1.05 (−1.45 to 3.55)	0.393	0.36 (−0.44 to 1.16)
12 months	71.68 ± 2.38ᶜ	71.48 ± 1.91ᶜ	0.20 (−1.62 to 2.02)	0.823	0.09 (−0.71 to 0.89)
*p* value (within group, over time)	<0.001*	<0.001*			

Different superscript lowercase letters (ᵃ, ᵇ, ᶜ) denote statistically significant differences between follow-up points within the same group (Bonferroni-adjusted *p* < 0.0167).

* Statistically significant at adjusted *α* = 0.05.

** Adjusted using Holm-Bonferroni correction for the three between-group comparisons at 3, 6, and 12 months.

For ISQ, the linear mixed-effects model revealed:

- Main effect of time: *F*_(2, 44)_ = 89.4, *p* < 0.001
- Main effect of group: *F*_(1, 11)_ = 2.3, *p* = 0.158
- Group × Time interaction: *F*_(2, 44)_ = 2.1, *p* = 0.135

Pairwise tests within groups were significant for most time-step comparisons (Table 3B), consistent with progressive osseointegration and maturation of the bone–implant interface irrespective of timing once standardized guided protocols and customized healing abutments are employed (18, 19).

**Table 3B:** Pairwise comparison regarding ISQ within each group.

Comparison	IIP *p*-value	EIP *p*-value
3 vs. 6 months	0.009*	<0.001*
3 vs. 12 months	<0.0001*	<0.001*
6 vs. 12 months	0.012*	<0.001*

* Adjusted using Bonferroni correction (*α* = 0.05/3 = 0.0167). Statistically significant at *p* < 0.0167.

### Esthetic outcomes (PES/WES)

#### Total scores

Aggregated soft-tissue esthetics (PES) favored IIP at both 6 months (8.67 ± 0.78 vs. 6.58 ± 1.00; mean difference: 2.09, 95% CI: 1.48–2.70; *p* < 0.001) and 12 months (8.42 ± 0.79 vs. 6.92 ± 1.00; mean difference: 1.50, 95% CI: 0.92–2.08; *p* < 0.001). Crown-related esthetics (WES) were similar at 6 months (8.75 ± 0.62 vs. 8.33 ± 0.89; mean difference: 0.42, 95% CI: −0.31 to 1.15; *p* = 0.234) and modestly favored IIP at 12 months (8.33 ± 0.65 vs. 7.33 ± 0.65; mean difference: 1.00, 95% CI: 0.50–1.50; *p* = 0.002) (Table 4A, Figure 7A).

**Table 4A:** Mean ± SD of total PES and WES at 6 and 12 months.

Parameter	Time	IIP (*n* = 12) mean ± SD	EIP (*n* = 12) mean ± SD	Mean difference (95% CI)	*p* value*	Effect size (Cohen’s *d*, 95% CI)
PES	6 months	8.67 ± 0.78	6.58 ± 1.00	2.09 (1.48 to 2.70)	<0.001*	2.35 (1.05 to 3.65)
	12 months	8.42 ± 0.79	6.92 ± 1.00	1.50 (0.92 to 2.08)	<0.001*	2.11 (0.85 to 3.37)
WES	6 months	8.75 ± 0.62	8.33 ± 0.89	0.42 (−0.31 to 1.15)	0.234	0.55 (−0.27 to 1.37)
	12 months	8.33 ± 0.65	7.33 ± 0.65	1.00 (0.50 to 1.50)	0.002*	1.54 (0.38 to 2.70)

* Adjusted using Holm-Bonferroni correction for the four comparisons (PES at 6 months, PES at 12 months, WES at 6 months, WES at 12 months). Statistically significant at adjusted *α* = 0.05.

For PES, the linear mixed-effects model revealed:

- Main effect of time: *F*_(1, 22)_ = 8.7, *p* = 0.007
- Main effect of group: *F*_(1, 11)_ = 45.6, *p* < 0.001
- Group × Time interaction: *F*_(1, 22)_ = 6.2, *p* = 0.021

#### Parameter-level analysis

For PES components, several soft-tissue parameters were more likely to attain the top category (“2”) with EIP at 12 months: soft-tissue level (50.0% vs. 8.3%; OR = 11.0, 95% CI: 1.06–114.3; *p* = 0.044), soft-tissue curvature (50.0% vs. 66.7%; OR = 0.50, 95% CI: 0.10–2.50; *p* = 0.034), and root convexity/color (66.7% vs. 91.7%; OR = 0.18, 95% CI: 0.02–1.87; *p* = 0.023). Mesial and distal papillae improved over time in both groups, with a shift toward higher category scores by 12 months (Table 4B). WES parameters were uniformly excellent for outline across all visits in both arms, and volume/axis, shade, and texture distributions progressed favorably over time without categorical differences (Table 4C). The esthetic panels and scores cases are illustrated in Figure 7B.

**Table 4B:** Frequency (percentage) of 5 PES parameters in immediate and early implant placement at 3, 6, and 12 months.

Parameter	Time	Group	Score 0, *n* (%)	Score 1, *n* (%)	Score 2, *n* (%)	*p* value
Mesial papilla (Mp)	3 months	Immediate (*n* = 12)	0 (0.0%)	0 (0.0%)	12 (100%)	0.241
		Early (*n* = 12)	0 (0.0%)	4 (33.3%)	8 (66.7%)	
	6 months	Immediate	0 (0.0%)	0 (0.0%)	12 (100%)	0.241
		Early	0 (0.0%)	4 (33.3%)	8 (66.7%)	
	12 months	Immediate	0 (0.0%)	11 (91.7%)	1 (8.3%)	0.024
		Early	0 (0.0%)	5 (41.7%)	7 (58.3%)	
Distal papilla (Dp)	3 months	Immediate	0 (0.0%)	10 (83.3%)	2 (16.7%)	0.211
		Early	0 (0.0%)	4 (33.3%)	8 (66.7%)	
	6 months	Immediate	0 (0.0%)	10 (83.3%)	2 (16.7%)	0.211
		Early	0 (0.0%)	3 (25.0%)	9 (75.0%)	
	12 months	Immediate	0 (0.0%)	0 (0.0%)	12 (100%)	0.500
		Early	0 (0.0%)	1 (8.3%)	11 (91.7%)	
Soft-tissue level (STL)	3 months	Immediate	0 (0.0%)	10 (83.3%)	2 (16.7%)	0.211
		Early	0 (0.0%)	4 (33.3%)	8 (66.7%)	
	6 months	Immediate	0 (0.0%)	10 (83.3%)	2 (16.7%)	0.066
		Early	2 (16.6%)	4 (33.3%)	6 (50.0%)^a^	
	12 months	Immediate	0 (0.0%)	11 (91.7%)	1 (8.3%)	0.044
		Early	1 (8.3%)	5 (41.6%)	6 (50.0%)^a^	
Soft-tissue curvature (STC)	3 months	Immediate	0 (0.0%)	3 (25.0%)	9 (75.0%)	0.143
		Early	3 (25.0%)	4 (33.3%)	5 (41.6%)	
	6 months	Immediate	0 (0.0%)	3 (25.0%)	9 (75.0%)	0.143
		Early	3 (25.0%)	4 (33.3%)	5 (41.6%)	
	12 months	Immediate	0 (0.0%)	4 (33.3%)	8 (66.7%)	0.034
		Early	1 (8.3%)	5 (41.6%)	6 (50.0%)^a^	
Root convexity/color (RC)	3 months	Immediate	0 (0.0%)	2 (16.7%)	10 (83.3%)	0.178
		Early	0 (0.0%)	4 (33.3%)	8 (66.7%)	
	6 months	Immediate	0 (0.0%)	2 (16.7%)	10 (83.3%)	0.178
		Early	0 (0.0%)	4 (33.3%)	8 (66.7%)	
	12 months	Immediate	0 (0.0%)	1 (8.3%)	11 (91.7%)	0.023
		Early	0 (0.0%)	4 (33.3%)	8 (66.7%)^a^	

*P*-values are unadjusted (exploratory analyses); interpret cautiously.

a McNemar’s test for paired binary data.

**Table 4C:** Frequency (percentage) of 5 WES parameters parameters in immediate and early implant placement at 3, 6, and 12 months.

Parameter	Time	Group	Score 0, *n* (%)	Score 1, *n* (%)	Score 2, *n* (%)	*p* value
Outline	3 months	Immediate (*n* = 12)	0 (0.0%)	0 (0.0%)	12 (100%)	1.000
		Early (*n* = 12)	0 (0.0%)	0 (0.0%)	12 (100%)	
	6 months	Immediate	0 (0.0%)	0 (0.0%)	12 (100%)	1.000
		Early	0 (0.0%)	0 (0.0%)	12 (100%)	
	12 months	Immediate	0 (0.0%)	0 (0.0%)	12 (100%)	1.000
		Early	0 (0.0%)	0 (0.0%)	12 (100%)	
Volume/Axis	3 months	Immediate	0 (0.0%)	0 (0.0%)	12 (100%)	1.000
		Early	0 (0.0%)	0 (0.0%)	12 (100%)	
	6 months	Immediate	0 (0.0%)	7 (58.3%)	5 (41.7%)	0.727
		Early	0 (0.0%)	6 (50.0%)	6 (50.0%)	
	12 months	Immediate	0 (0.0%)	5 (41.7%)	7 (58.3%)	1.000
		Early	0 (0.0%)	5 (41.7%)	7 (58.3%)	
Shade	3 months	Immediate	0 (0.0%)	0 (0.0%)	12 (100%)	1.000
		Early	0 (0.0%)	0 (0.0%)	12 (100%)	
	6 months	Immediate	0 (0.0%)	5 (41.7%)	7 (58.3%)	1.000
		Early	0 (0.0%)	5 (41.7%)	7 (58.3%)	
	12 months	Immediate	0 (0.0%)	6 (50.0%)	6 (50.0%)	1.000
		Early	1 (8.3%)	5 (41.7%)	6 (50.0%)	
Texture	3 months	Immediate	0 (0.0%)	0 (0.0%)	12 (100%)	1.000
		Early	0 (0.0%)	0 (0.0%)	12 (100%)	
	6 months	Immediate	2 (16.7%)	5 (41.7%)	5 (41.7%)	1.000
		Early	2 (16.7%)	5 (41.7%)	5 (41.7%)	
	12 months	Immediate	0 (0.0%)	6 (50.0%)	6 (50.0%)	1.000
		Early	1 (8.3%)	5 (41.7%)	6 (50.0%)	
Translucency	3 months	Immediate	0 (0.0%)	0 (0.0%)	12 (100.0%)	1.000
		Early	0 (0.0%)	0 (0.0%)	12 (100.0%)	
	6 months	Immediate	0 (0.0%)	5 (41.7%)	7 (58.3%)	1.000
		Early	0 (0.0%)	5 (41.7%)	7 (58.3%)	
	12 months	Immediate	0 (0.0%)	6 (50.0%)	6 (50.0%)	1.000
		Early	1 (8.3%)	5 (41.7%)	6 (50.0%)	

### Peri-implant health parameters

Clinical peri-implant conditions trended toward healthier profiles over time in both strategies. Probing-depth (PD) frequency distributions shifted away from 3-mm readings toward greater proportions of 1–2 mm at both labial and palatal sites by 12 months (Table 5A, Figure 8). Within-group pairwise testing showed significant PD improvement for IIP from 3 → 6 months (*p* = 0.000010) and 3 → 12 months (*p* = 0.00004), while analogous changes for EIP were not statistically significant for those intervals (*p* = 0.36 and *p* = 0.387, respectively); the 6 → 12-month comparison was non-significant in both arms (Table 5B). Bleeding scores remained low and comparable between groups at all timepoints (all *p* ≥ 0.657, paired *t*-tests with Holm-Bonferroni correction. Within-group changes over time were not statistically significant for either group (all *p* ≥ 0.8389, repeated-measures ANOVA with Greenhouse-Geisser correction). The effect sizes for between-group BI differences were consistently small (Cohen's *d*: 0.02–0.26), indicating clinically negligible differences in bleeding tendency between timing strategies (Table 5C, Figure 9).

**Table 5A:** Probing depth (PD) frequency (percentage) at labial and palatal sites (scores = 1, 2, or 3 mm) at 3, 6, and 12 months.

Site	Time	Group	1 mm *n* (%)	2 mm *n* (%)	3 mm *n* (%)	*p* value
Labial	3 m	Immediate (*n* = 12)	0 (0.0%)	0 (0.0%)	12 (100%)	0.092
		Early (*n* = 12)	2 (16.6%)	8 (66.6%)	2 (16.6%)	
	6 m	Immediate	2 (16.6%)	8 (66.6%)	2 (16.6%)	0.037*
		Early	2 (16.6%)	9 (75.0%)	1 (8.3%)	
	12 m	Immediate	5 (41.6%)	4 (33.3%)	3 (25.0%)	0.090
		Early	4 (33.3%)	5 (41.6%)	3 (25.0%)	
Palatal	3 m	Immediate	0 (0.0%)	3 (25.0%)	9 (75.0%)	0.084
		Early	3 (25.0%)	7 (58.3%)	2 (16.6%)	
	6 m	Immediate	0 (0.0%)	4 (33.3%)	8 (66.7%)	0.090
		Early	4 (33.3%)	5 (41.6%)	3 (25.0%)	
	12 m	Immediate	1 (8.3%)	5 (41.6%)	6 (50.0%)	0.064
		Early	5 (41.6%)	5 (41.6%)	2 (16.6%)	

3 m, 3 months; 6 m, 6 months; 12 m, 12 months.

* *p* < 0.05 statistically significant.

**Table 5B:** Pairwise comparison regarding probing depth (mm) within each group.

Comparison	IIP *p*-value	EIP *p*-value
3 vs. 6 months	0.00001*	0.360
3 vs. 12 months	0.00004*	0.387
6 vs. 12 months	0.290	0.500

* Adjusted using Bonferroni correction (*α* = 0.05/3 = 0.0167). Statistically significant at *p* < 0.0167.

**Table 5C:** Modified sulcus bleeding index (BI) at 3, 6, and 12 months (means ± SD).

Site/Time	IIP mean ± SD	EIP mean ± SD	*p* value*	Effect size (Cohen’s *d*, 95% CI)
Labial at 3 months	0.50 ± 0.52	0.58 ± 0.51	0.682	0.16 (−0.64 to 0.96)
Palatal at 3 months	0.58 ± 0.51	0.67 ± 0.49	0.673	0.18 (−0.62 to 0.98)
Labial at 6 months	0.52 ± 0.48	0.53 ± 0.49	0.672	0.02 (−0.78 to 0.82)
Palatal at 6 months	0.51 ± 0.58	0.51 ± 0.67	0.657	0.00 (−0.80 to 0.80)
Labial at 12 months	0.52 ± 0.49	0.54 ± 0.52	0.680	0.04 (−0.76 to 0.84)
Palatal at 12 months	0.49 ± 0.53	0.50 ± 0.54	0.684	0.02 (−0.78 to 0.82)

* Adjusted using Holm-Bonferroni correction for the six between-group comparisons.

**Table 5D:** Pairwise comparison regarding bleeding index within each group.

Comparison	IIP *p*-value*	EIP *p*-value*
3 vs. 6 months	0.839	0.923
3 vs. 12 months	0.851	0.924
6 vs. 12 months	0.962	1.000

* Adjusted using Bonferroni correction (*α* = 0.05/3 = 0.0167). No significant differences found.

**Table 6:** Observed power and precision for key outcomes.

Outcome	Observed difference (95% CI)	Effect size (*d*)	Power achieved (for observed difference)^a^
MBL at 12 months	−0.01 (−0.08 to 0.06)	0.15	8%
ISQ at 12 months	0.20 (−1.62 to 2.02)	0.09	6%
PES at 12 months	1.50 (0.92 to 2.08)	2.11	99%
WES at 12 months	1.00 (0.50 to 1.50)	1.54	97%

a Power calculations based on paired *t*-test with *n* = 12, *α* = 0.05 (two-tailed). Power for MBL and ISQ is low because the observed differences are very small; the study was powered to detect a clinically meaningful difference (0.25 mm), not the small differences actually observed.

Collectively, these peri-implant indices align with the convergent hard- and soft-tissue stability observed at 1 year and suggested that the standardized maintenance protocol supported favorable tissue conditions in both timing strategies.

In summary, the dataset shows (i) similar 12-month marginal bone outcomes with either timing, (ii) earlier stability signals and lower early MBL with IIP, (iii) strong esthetic performance across protocols with parameter-level soft-tissue advantages detectable in EIP at 12 months but higher total PES and 12-month WES in IIP, and (iv) broadly healthy peri-implant tissue profiles with low bleeding and improving probing depths over time (Tables 5D, 6). These findings, taken with the standardized guided workflow and consistent transmucosal contouring via customized healing abutments, underscore that both approaches can meet functional and esthetic objectives in the anterior zone, with clinically relevant trade-offs in early remodeling and specific soft-tissue contours that may guide individualized timing decisions (13, 20).

## Discussion

The present randomized split-mouth clinical trial evaluated the 12-month outcomes of immediate vs. early anterior implant placement when both protocols employed Cervico-designed customized healing abutments to guide peri-implant soft-tissue maturation. By using a split-mouth design, patient-related confounders, including bone quality, soft-tissue phenotype, occlusal patterns, and healing capacity, were controlled, allowing a more precise comparison of the two timing protocols. The rationale for selecting a 4-week interval for early implant placement was to allow soft-tissue closure and initial healing while preserving ridge architecture. Immediate placement was included to evaluate its potential advantages in preserving socket anatomy and reducing treatment time. The findings demonstrated comparable marginal bone loss (MBL), implant stability, and esthetic outcomes between immediate and early implants, although subtle differences highlight the clinical considerations for each approach (20).

The split-mouth design strengthened the internal validity of the study, minimizing patient-level variability and allowing direct comparison of the two protocols under identical biological conditions. This design is particularly advantageous in esthetic-zone implant research, where individual anatomical differences can heavily influence outcomes. Nevertheless, it also introduces potential for cross-arch biological influence, although such effects are minimal in implant dentistry (18).

### Marginal bone loss

Both immediate and early implants exhibited minimal marginal bone after 12 months, consistent with contemporary literature supporting the predictability of both placement timings in the esthetic zone. The absence of a statistically significant difference at 12 months (mean difference: −0.01 mm, 95% CI: −0.08 to 0.06) suggests that any true difference is likely small and clinically negligible. However, it should be noted that the 95% confidence interval for the between-group difference did not include values exceeding the pre-specified clinically meaningful threshold of 0.25 mm, supporting the conclusion of clinical equivalence. The narrow confidence interval indicates that the study had adequate precision to rule out a clinically important difference, despite the relatively small sample size. These findings suggest that when case selection is appropriate and a proper soft-tissue scaffold is provided, immediate placement does not inherently increase the risk of bone loss beyond clinically acceptable limits (19).

Immediate placement has traditionally been associated with potential remodeling risks due to the presence of the buccal plate and residual socket biology. However, the use of customized healing abutments likely mitigated this by stabilizing the flapless environment and promoting a predictable emergence profile that preserved peri-implant tissue contours (12).

The statistical significance of MBL reductions within both groups over time, underscored by *p*-values less than 0.001, further corroborates the phenomenon of early post-implantation bone remodelling. Such findings are in harmony with the body of literature that emphasizes the criticality of the initial months post-implantation for bone healing and adaptation. This period appears to be pivotal for the establishment of a stable bone-implant interface, which subsequently evolves into a less dynamic, more stabilized state, minimizing bone loss as the implant integrates with the surrounding bone matrix (21).

### Implant stability

Implant stability increased over time in both protocols; IIP demonstrated higher ISQ at the 3-month assessment, with convergence by 6 and 12 months. This aligns with biomechanical principles, as early placement allows partial resolution of inflammatory tissue and initial bone fill that may enhance insertion torque and resonance frequency measurements (12). Nonetheless, immediate implants still achieved sufficient stability for safe functional loading, likely aided by apical engagement beyond the socket and careful implant selection (21).

### Esthetic outcomes

Esthetic scores were high in both groups; however, total PES favored IIP at 6 and 12 months, and WES favored IIP at 12 months, while several PES subcomponents (e.g., soft-tissue level/curvature and root convexity/color) favored EIP at 12 months (22). The superior soft-tissue esthetic outcomes observed with IIP are likely attributable to the timing of implant placement rather than the customized healing abutments *per se*. Immediate placement allows preservation of the natural socket architecture and soft-tissue contours, reducing the volumetric changes that typically occur following tooth extraction. The customized healing abutments, however, likely contributed to the favorable outcomes observed in both groups by providing a tailored transmucosal scaffold that guided soft-tissue maturation and maintained the emergence profile. This interpretation is supported by previous studies demonstrating that anatomical healing abutments improve peri-implant tissue conditioning regardless of placement timing. The standardized use of customized abutments in both arms controlled for this variable, allowing the true effect of timing to be isolated (23).

The results support the use of immediate placement, even in the anterior esthetic zone, as a predictable option when combined with customized healing abutments and proper case selection. Immediate placement offers advantages such as reduced treatment time, fewer surgical interventions, and early preservation of soft-tissue contours. Conversely, early placement remains a valuable alternative when immediate insertion is contraindicated, such as in the presence of thin buccal bone, acute infection, or insufficient primary stability. The similarity in outcomes suggests that clinicians may choose between the two-timing strategies based on patient anatomy, risk profile, and preference, with reassurance that both approaches can produce stable and esthetic results when supported by customized soft-tissue management (24).

### Guided bone regeneration

While the present study focused on sites with adequate bone volume (≥4 mm apical bone and ≥2 mm labial plate), it is important to acknowledge that guided bone regeneration (GBR) represents a key prerequisite for successful implant placement in areas with inadequate bone volume. GBR techniques enable vertical and horizontal augmentation of atrophic alveolar ridges, facilitating implant placement in the correct three-dimensional position and ensuring optimal esthetic outcomes. Whether performed simultaneously with implant placement or as a staged procedure prior to implantation, GBR has demonstrated excellent clinical and esthetic results in the rehabilitation of compromised sites (25). These regenerative approaches are particularly valuable when immediate or early placement is contraindicated due to insufficient bone volume, and they complement the customized healing abutment protocols described in the present study.

The study demonstrated several statistical strengths that enhance the validity of its findings. The split-mouth design minimized patient-related confounding, while mixed-effects models appropriately accounted for the paired repeated-measures data. Standardized CBCT imaging protocols reduced measurement variability, resulting in precise marginal bone loss (MBL) estimates. Additionally, the use of two one-sided tests (TOST) for equivalence provided stronger evidence of clinical comparability than conventional hypothesis testing, and the complete follow-up (100% retention) eliminated the risk of attrition bias.

The findings also highlight the importance of distinguishing statistical significance from clinical relevance. Although no significant difference was observed in 12-month MBL between groups (*p* = 0.618), the small effect size (*d* = 0.15) and narrow 95% confidence interval, which remained well below the predefined clinically meaningful threshold of 0.25 mm, indicate that any true difference is unlikely to be clinically relevant. In contrast, the significantly higher Pink Esthetic Scores (PES) in the immediate implant placement group (*p* < 0.001) were associated with large effect sizes (*d* = 2.09–2.11), demonstrating both statistical and clinical significance. The approximately 1.5-point improvement in PES, corresponding to a 15% enhancement in esthetic outcomes, represents a meaningful benefit for anterior implant rehabilitation.

### Limitations

Several limitations should be considered when interpreting these findings. Although the sample size was adequately powered for the primary outcome, the relatively small, single-center cohort may limit the generalizability of the results and the ability to detect subtle differences in secondary outcomes. Furthermore, the study population was restricted to young adults with thick soft-tissue phenotype and favorable bone conditions in the anterior maxilla, limiting extrapolation to patients with thin tissue biotypes, compromised sites, or other anatomical regions. The 12-month follow-up was sufficient to assess early bone remodeling and esthetic outcomes but does not provide evidence of long-term stability. In addition, the use of a single implant system, guided surgical protocol, and customized healing abutments in both groups limits the applicability of the findings to other clinical approaches and precludes assessment of the independent effect of customized abutments. Future multicenter studies with larger, more diverse populations, longer follow-up periods, and appropriate control groups are warranted to validate these findings. Nevertheless, the split-mouth design, standardized treatment protocols, and complete follow-up provide strong internal validity and support the reliability of the comparison between immediate and early implant placement. The results are consistent with previous systematic reviews showing that immediate implants, when performed under controlled conditions, yield comparable bone and functional outcomes to early or delayed approaches. The added value of customized healing abutments, increasingly supported by digital workflows, is now gaining evidence as a key factor in soft-tissue preservation. This trial reinforces the notion that the prosthetically driven, minimally disrupted soft-tissue interface is fundamental for long-term stability (25–27).

## Conclusion

Taken together, immediate and early anterior implant placement present similarly favorable outcomes in terms of bone remodeling and stability over 12 months. Formal equivalence testing confirmed that the two timing strategies produce clinically comparable marginal bone outcomes. However, overall soft-tissue esthetics (PES) favored immediate placement, while specific soft-tissue parameters favored early placement at 12 months, indicating a nuanced esthetic trade-off when customized healing abutments are used. The results support the incorporation of personalized transmucosal components into contemporary anterior implant protocols and underscore the importance of biologically oriented peri-implant tissue management in esthetically demanding sites.

## Data Availability

The raw data supporting the conclusions of this article will be made available by the authors, without undue reservation.
